# New contributions to Diatrypaceae from karst areas in China

**DOI:** 10.3897/mycokeys.83.68926

**Published:** 2021-08-20

**Authors:** Sihan Long, Lili Liu, Yinhui Pi, Youpeng Wu, Yan Lin, Xu Zhang, Qingde Long, Yingqian Kang, Jichuan Kang, Nalin N. Wijayawardene, Feng Wang, Xiangchun Shen, Qirui Li

**Affiliations:** 1 State Key Laboratory of Functions and Applications of Medicinal Plants, Guizhou Medical University, Guiyang 550004, China; 2 The High Efficacy Application of Natural Medicinal Resources Engineering Center of Guizhou Province (The Key Laboratory of Optimal Utilization of Natural Medicine Resources), School of Pharmaceutical Sciences, Guizhou Medical University, University Town, Guian New District, Guizhou 550025, China; 3 Immune Cells and Antibody Engineering Research Center of Guizhou Province/ Key Laboratory of Biology and Medical Engineering, Guizhou Medical University, Guiyang 550004, China; 4 Key Laboratory of Environmental Pollution Monitoring and Disease Control, Ministry of Education of Guizhou and Guizhou Talent Base for Microbiology and Human Health, School of Basic Medical Sciences, Guizhou Medical University, Guiyang, China; 5 Engineering and Research Center for Southwest Bio-Pharmaceutical Resources of National Education Ministry of China, Guizhou University, Guiyang, Guizhou 550025, China; 6 Center for Yunnan Plateau Biological Resources Protection and Utilization, College of Biological Resource and Food Engineering, Qujing Normal University, Qujing, Yunnan 655011, China; 7 Guizhou Provincial Academician Workstation of Microbiology and Health, Guizhou Academy of Tobacco Science, Guiyang, Guizhou, 550000, China

**Keywords:** Five novel taxa, phylogeny, systematics, taxonomy, Xylariales

## Abstract

In this study, fungal specimens of the family Diatrypaceae were collected from karst areas in Guizhou, Hainan and Yunnan Provinces, China. Morpho-molecular analyses confirmed that these new collections comprise a new genus *Pseudodiatrype*, three new species (*Diatrypelancangensis*, *Diatrypellapseudooregonensis* and *Eutypacerasi*), a new combination (*Diatrypellaoregonensis*), two new records (*Allodiatrypethailandica* and *Diatrypellavulgaris*) from China and two other known species (*Neoeutypellabaoshanensis* and *Paraeutypellacitricola*). The new taxa are introduced, based on multi-gene phylogenetic analyses (ITS, β-tubulin), as well as morphological analyses. The new genus *Pseudodiatrype* is characterised by its wart-like stromata with 5–20 ascomata immersed in one stroma and the endostroma composed of thin black outer and inner layers of large white cells with thin, powdery, yellowish cells. These characteristics separate this genus from two similar genera *Allodiatrype* and *Diatrype*. Based on morphological as well as phylogenetic analyses, *Diatrypelancangensis* is introduced as a new species of *Diatrype*. The stromata of *Diatrypelancangensis* are similar to those of *D.subundulata* and *D.undulate*, but the ascospores are larger. Based on phylogenetic analyses, *Diatrypeoregonensis* is transferred to the genus *Diatrypella* as *Diatrypellaoregonensis* while *Diatrypellapseudooregonensis* is introduced as a new species of *Diatrypella* with 8 spores in an ascus. In addition, multi-gene phylogenetic analyses show that *Eutypacerasi* is closely related to *E.lata*, but the ascomata and asci of *Eutypacerasi* are smaller. The polyphyletic nature of some genera of Diatrypaceae has led to confusion in the classification of the family, thus we discuss whether the number of ascospores per asci can still be used as a basis for classification.

## Introduction

Diatrypaceae is an important family of higher ascomycetes, belonging to Xylariales (Maharachchikumbura et al. 2016). In the latest compilation, Hyde et al. (2020a) revised the family Diatrypaceae and included several new genera (i.e. *Allodiatrype* Konta & K.D. Hyde, *Halocryptovalsa* Dayar. & K.D. Hyde and *Neoeutypella* M. Raza et al.). This was followed by Wijayawardene et al. (2020) in which 20 genera were accepted into Diatrypaceae. The Diatrypaceae is characterised by perithecial ascomata embedded in a poor or well-developed, brown or black-coloured stroma, long-stalked and 8-spored or numerous-spored asci and allantoid, unicellular ascospores (Glawe and Rogers 1984; Rappaz 1987; Mehrabi et al. 2015; de Almeida et al. 2016).

Members of Diatrypaceae occur on a wide range of hosts in terrestrial and marine environments worldwide, some of which are important plant pathogens (Moyo et al. 2018a; Mehrabi et al. 2019; Dayarathne et al. 2020; Konta et al. 2020). For many decades, canker diseases on grapevine have been attributed to the species of Diatrypaceae worldwide, for example in China *Cryptovalsa* Ces. & De Not., *Cryptosphaeria* Ces. & De Not, *Diatrype* Fr., *Diatrypella* (Ces. & De Not.) De Not., *Eutypa* Tul. & C. Tul. And *Eutypella* (Nitschke) Sacc., are responsible for canker diseases in grapevine (Trouillas et al. 2011; Gao et al. 2013; Moyo et al. 2018b). Besides cankers of grapevine, some species have been reported as the causal pathogentic agents of fruit trees and woody plants in Europe and the USA (Trouillas et al. 2011; Gao et al. 2013).

Thirteen species of *Cryptosphaeria* and *Diatrype* were introduced by Vasiljeva and Ma (2014) from north-eastern China, which includes two new species and four new records. China has the largest range of karst distribution in the world. The landform of karst can be found in almost all Provinces of China, with the most extensive distribution in Guizhou and Yunnan Provinces (Miao et al. 2007). Karst virgin forest is a relatively stable ecosystem with rich biological resources, highly primitive and maintaining stable biological diversity (Dong et al. 2002). The special karst and ecological environment is home to a rich diversity of diatrypaceous fungi.

In this study, we revisit species of Diatrypaceae collected from karst areas in Guizhou, Hainan and Yunnan Provinces of China. Based on morpho-molecular analyses, one new genus and three new species are introduced; in addition, a new combination and two new records from China are reported. Descriptions and illustrations of new taxa and new records are provided.

## Materials and Methods

### Fungi collection, isolation and identification

Samples of decaying wood were collected from October 2019 to November 2020 in forests and nature reserves of Guizhou, Hainan and Yunnan Provinces in China. The specimens were observed with a stereomicroscope while microscopic images of the samples were taken using a Nikon ECLIPSE Ni compound microscope, with a Canon EOS 700D digital camera. Measurements were taken with Tarosoft (R) Image Frame Work (v.0.9.7). More than 30 asci and ascospores were measured for each specimen examined. Photoplates were arranged and improved by using Adobe Photoshop CS6 software. Isolations of fungi were made by single spore isolation (Chomnunti et al. 2014) and germinated spores were transferred to potato dextrose agar (**PDA**) medium for purification. The specimens were deposited at the Herbarium of Cryptogams, Kunming Institute of Botany Academia Sinica (**KUN-HKAS**) and Herbarium of Guizhou Medical University (**GMB**). Strains of the new genus and new species are maintained in the Guizhou Medical University Collection Centre (**GMBC**).

**Table 1.** Taxa used in the phylogenetic analyses and their corresponding GenBank accession numbers.

Taxa	Strain number	GenBank Accession number		Reference
		ITS	β-tubulin	
* Allocryptovalsa elaeidis *	MFLUCC 15-0707	MN308410	MN340296	Konta et al. (2020)
* A. polyspora ^T^*	MFLUCC 17-0364	MF959500	MG334556	Senwanna et al. (2017)
* A. rabenhorstii *	WA08CB	HQ692619	HQ692523	Trouillas et al. (2011)
* Allodiatrype arengae ^T^*	MFLUCC 15-0713	MN308411	MN340297	Konta et al. (2020)
* A. elaeidicola *	MFLUCC 15-0737a	MN308415	MN340299	Konta et al. (2020)
* A. elaeidis *	MFLUCC 15-0708a	MN308412	MN340298	Konta et al. (2020)
* A. thailandica *	MFLUCC 15-3662	KU315392	NA	Li et al. (2016)
* A. thailandica *	MFLUCC 15-0711	MN308414	NA	Konta et al. (2020)
*** A. thailandica ***	**GMB0050**	**MW797108**	**MW814880**	This study
* Anthostoma decipiens ^T^*	IPV-FW349	AM399021	AM920693	Unpublished.
* A. decipiens ^T^*	JL567	JN975370	JN975407	Luque et al. (2012)
* Cryptosphaeria ligniota *	CBS 273.87	KT425233	KT425168	Acero et al. (2004)
* C. pullmanensis *	ATCC 52655	KT425235	KT425170	Trouillas et al. (2015)
* C. subcutanea *	DSUB100A	KT425189	KT425124	Trouillas et al. (2015)
* C. subcutanea *	CBS 240.87	KT425232	KT425167	Trouillas et al. (2015)
* Cryptovalsa ampelina *	A001	GQ293901	GQ293972	Trouillas et al. (2010)
* C. ampelina *	DRO101	GQ293902	GQ293982	Trouillas et al. (2010)
* Diatrype bullata *	UCDDCh400	DQ006946	DQ007002	Rolshausen et al. (2006)
* D. disciformis ^T^*	GNA14	KR605644.1	KY352434.1	Senanayake et al. (2015)
* D. disciformis ^T^*	D21C, CBS 205.87	AJ302437	NA	Acero et al. (2004)
* D. enteroxantha *	HUEFS155114	KM396617	KT003700	de Almeida et al. (2016)
* D. enteroxantha *	HUEFS155116	KM396618	KT022236	de Almeida et al. (2016)
*** D. lancangensis ***	**GMB0045**	**MW797113**	**MW814885**	**This study**
*** D. lancangensis ***	**GMB0046**	**MW797114**	**MW814886**	**This study**
*** D. lancangensis ***	**GMB0047**	**MW797116**	**MW814887**	**This study**
* D. palmicola *	MFLUCC 11-0020	KP744438	NA	Liu et al. (2015)
* D. palmicola *	MFLUCC 11-0018	KP744439	NA	Liu et al. (2015)
* D. spilomea *	D17C	AJ302433	NA	Acero et al. (2004)
* D. stigma *	DCASH200	GQ293947	GQ294003	Trouillas et al. (2010)
* D. undulata *	D20C, CBS 271.87	AJ302436	NA	Acero et al. (2004)
* Diatrypella atlantica *	HUEFS 136873	KM396614	KR259647	de Almeida et al. (2016)
* D. banksiae *	CPC 29118	KY173402	NA	Crous et al. (2013)
* D. delonicis *	MFLUCC 15-1014	MH812994	MH847790	Hyde et al. (2019)
* D. delonicis *	MFLU 16-1032	MH812995	MH847791	Hyde et al. (2019)
* D. elaeidis *	MFLUCC 15-0279	MN308417	MN340300	Konta et al. (2020)
* D. favacea *	Islotate 380	KU320616	NA	de Almeida et al. (2016)
* D. favacea *	DL26C	AJ302440	NA	Unpublished
* D. frostii *	UFMGCB 1917	HQ377280	NA	Vieira et al. (2011)
* D. heveae *	MFLUCC 15-0274	MN308418	MN340301	Konta et al. (2020)
* D. heveae *	MFLUCC 17-0368	MF959501	MG334557	Senwanna et al. (2017)
* D. hubeiensis *	CFCC 52413	MW632937	NA	Zhu et al. (2021)
* D. iranensis *	KDQ18	KM245033	KY352429	Mehrabi et al. (2015)
* D. macrospora *	KDQ15	KR605648	KY352430	Mehrabi et al. (2016)
*D.oregonensis* (*Diatrypeoregonensis*)	DPL200	GQ293940	GQ293999	Trouillas et al. (2010)
*D.oregonensis* (*Diatrypeoregonensis*)	CA117	GQ293934	GQ293996	Trouillas et al. (2010)
*** D. pseudooregonensis ***	**GMB0039**	**MW797115**	**MW814888**	**This study**
*** D. pseudooregonensis ***	**GMB0040**	**MW797117**	**MW814889**	**This study**
*** D. pseudooregonensis ***	**GMB0041**	**MW797118**	**MW814890**	**This study**
*** D. pseudooregonensis ***	**GMB0042**	**MW797119**	**MW814891**	**This study**
*** D. pseudooregonensis ***	**GMB0043**	**MW797120**	**MW814892**	**This study**
*** D. pseudooregonensis ***	**GMB0044**	**MW797110**	**MW814882**	**This study**
* D. pulvinata *	H048	FR715523	FR715495	de Almeida et al. (2016)
* D. pulvinata *	DL29C	AJ302443	NA	Unpublished
* D. tectonae *	MFLUCC 12-0172a	KY283084	NA	Shang et al. (2017)
* D. tectonae *	MFLUCC 12-0172b	KY283085	KY421043	Shang et al. (2017)
* D. verruciformis ^T^*	UCROK1467	JX144793	JX174093	Lynch et al. (2013)
* D. verruciformis ^T^*	UCROK754	JX144783	JX174083	Lynch et al. (2013)
* D. vulgaris *	HVFRA02	HQ692591	HQ692503	Trouillas et al. (2011)
* D. vulgaris *	HVGRF03	HQ692590	HQ692502	Trouillas et al. (2011)
*** D. vulgaris ***	**GMB0051**	**MW797107**	**MW814879**	**This study**
* D. yunnanensis *	VT01	MN653008	MN887112	Zhu et al. (2021)
* Eutypa armeniacae *	ATCC 28120	DQ006948	DQ006975	Rolshausen et al. (2006)
* E. astroidea *	E49C, CBS 292.87	AJ302458	DQ006966	Rolshausen et al. (2006)
*** E. cerasi ***	**GMB0048**	**MW797104**	**MW814893**	**This study**
*** E. cerasi ***	**GMB0049**	**MW797105**	**MW814877**	**This study**
* E. flavovirens *	E48C, CBS 272.87	AJ302457	DQ006959	Rolshausen et al. (2006)
* E. laevata *	E40C CBS 291.87	AJ302449	NA	Acero et al. (2004)
* E. lata ^T^*	CBS290.87	HM164736	HM164770	Trouillas and Gubler (2010)
* E. lata ^T^*	EP18	HQ692611	HQ692501	Trouillas et al. (2011)
* E. lata ^T^*	RGA01	HQ692614	HQ692497	Trouillas et al. (2011)
* E. leioplaca *	CBS 248.87	DQ006922	DQ006974	Rolshausen et al. (2006)
* E. leptoplaca *	CBS 287.87	DQ006924	DQ006961	Rolshausen et al. (2006)
* E. maura *	CBS 219.87	DQ006926	DQ006967	Rolshausen et al. (2006)
* E. microasca *	BAFC 51550	KF964566	KF964572	Grassi et al. (2014)
* E. sparsa *	3802 3b	AY684220	AY684201	Trouillas and Gubler (2004)
* E. tetragona *	CBS 284.87	DQ006923	DQ006960	Rolshausen et al. (2006)
* Eutypella caricae *	EL51C	AJ302460	NA	Acero (2000)
* E. cerviculata ^T^*	M68	JF340269	NA	Arhipova et al. (2012)
* E. cerviculata ^T^*	EL59C	AJ302468	NA	Acero et al. (2004)
* E. leprosa *	EL54C	AJ302463	NA	Acero et al. (2004)
* E. leprosa *	Isolate 60	KU320622	NA	de Almeida et al. (2016)
* E. microtheca *	BCMX01	KC405563	KC405560	Paolinelli-Alfonso et al. (2015)
* E. parasitica *	CBS 210.39	DQ118966	NA	Jurc et al. (2006)
* E. semicircularis *	MP4669	JQ517314	NA	Mehrabi et al. (2016)
* Halocryptovalsa salicorniae *	MFLUCC 15-0185	MH304410	MH370274	Dayarathne et al. (2020)
* Halodiatrype avicenniae *	MFLUCC 15-0953	KX573916	KX573931	Dayarathne et al. (2016)
* H. salinicola ^T^*	MFLUCC 15-1277	KX573915	KX573932	Dayarathne et al. (2016)
* Kretzschmaria deusta *	CBS 826.72	KU683767	KU684190	U’Ren et al. (2016)
* Monosporascus cannonballus * ***^T^***	CMM3646	JX971617	NA	Unpublished
* M. cannonballus * ***^T^***	ATCC 26931	FJ430598	NA	Unpublished
*** Neoeutypella baoshanensis ^T^***	**GMB0052**	**MW797106**	**MW814878**	**This study**
* N. baoshanensis ^T^*	LC 12111	MH822887	MH822888	Hyde et al. (2019)
* N. baoshanensis ^T^*	EL51C, CBS 274.87	AJ302460	NA	Acero et al. (2004)
* N. baoshanensis ^T^*	MFLUCC 16-1002	MT310662	NA	Phukhamsakda et al. (2020)
* N. baoshanensis ^T^*	GL08362	JX241652	NA	Gao et al.(2013)
* Paraeutypella citricola *	HVVIT07	HQ692579	HQ692512	Trouillas et al. (2011)
* Pa. citricola *	HVGRF01	HQ692589	HQ692521	Trouillas et al. (2011)
*** Pa. citricola ***	**GMB0053**	**MW797109**	**MW814881**	This study
* Pa. guizhouensis ^T^*	KUMCC 20-0016	MW039349	MW239660	Dissanayake et al. (2021)
* Pa. guizhouensis ^T^*	KUMCC 20-0017	MW036141	MW239661	Dissanayake et al. (2021)
* Pa. vitis *	UCD2291AR	HQ288224	HQ288303	Úrbez-Torres et al. (2012)
* Pa. vitis *	UCD2428TX	FJ790851	GU294726	Úrbez-Torres et al. (2009)
* Pedumispora rhizophorae ^T^*	BCC44877	KJ888853	NA	Klaysuban et al. (2014)
* Pe. rhizophorae ^T^*	BCC44878	KJ888854	NA	Klaysuban et al. (2014)
* Peroneutypa alsophila *	EL58C, CBS 250.87	AJ302467	NA	Acero et al. (2004)
* Pe. curvispora *	HUEFS 136877	KM396641	NA	de Almeida et al. (2016)
* Pe. diminutispora *	MFLUCC 17-2144	MG873479	NA	Shang et al. (2018)
* Pe. mackenziei *	MFLUCC 16-0072	KY283083	KY706363	Shang et al. (2017)
* Pe. mangrovei *	PUFD526	MG844286	MH094409	Phookamsak et al. (2019)
*** Pseudodiatrype hainanensis ^T^***	**GMB0054**	**MW797111**	**MW814883**	**This study**
*** Ps. hainanensis ^T^***	**GMB0055**	**MW797112**	**MW814884**	**This study**
* Quaternaria quaternata *	EL60C, CBS 278.87	AJ302469	NA	Acero et al. (2004)
* Q. quaternata *	GNF13	KR605645	NA	Mehrabi et al. (2016)
* Xylaria hypoxylon *	CBS 122620	AM993141	KX271279	Peršoh et al. (2009)

**^T^**: Types species of the genus; **NA**: No sequence is available in GenBank; Newly generated sequences are indicated in **bold**.

### DNA extraction, Polymerase Chain Reaction (PCR) and phylogenetic analyses

Genomic DNA was extracted from fungal mycelium following the manufacturer’s protocol of the BIOMIGA Fungal gDNA isolation Kit (BIOMIGA, Hangzhou City, Zhejiang Province, China). Extracts of DNA were stored at –20 °C.

PCR was carried out in a volume of 25 μl containing 9.5 μl of ddH_2_O, 12.5 μl of 2× Taq PCR Master Mix (2 × Taq Master Mix with dye, TIANGEN, China), 1 μl of DNA extracts and 1 μl of forward and reverse primers (10 μM each) in each reaction. Primers pairs, ITS4 and ITS5, fRPB2-7CR and fRPB2-5f, LROR and LR5, T1 and Bt2b, as well as Bt2a and Bt2b (Vilgalys and Hester 1990; White et al. 1990; Glass and Donaldson 1995; O’Donnell and Cigelnik 1997), were used to amplify internal transcribed spacer (ITS) sequences, RNA polymerase II second largest subunit (RPB2) sequences, large subunit ribosomal (LSU) sequences and β-tubulin (TUB2) sequences, respectively.

PCR profiles for the ITS and LSU are as follows: initially at 95 °C for 5 minutes, followed by 35 cycles of denaturation at 94 °C for 1 minute, annealing at 52 °C for 1 minute, elongation at 72 °C for 1.5 minutes and a final extension at 72 °C for 10 minutes. PCR profile for the RPB2 is as follows: initially at 95 °C for 5 minutes, followed by 35 cycles of denaturation at 95 °C for 1 minute, annealing at 54 °C for 2 minutes, elongation at 72 °C for 1.5 minutes and a final extension at 72 °C for 10 minutes (Konta et al. 2020). PCR profile for the TUB2 are as follows: initially at 95 °C for 5 minutes, followed by 35 cycles of denaturation at 94 °C for 1 minute, annealing at 52 °C for 1 minute, elongation at 72 °C for 1.5 minutes and a final extension at 72 °C for 10 minutes (de Almeida et al. 2016). PCR products were submitted to Sangon Biotech, Shanghai, China for purification and sequencing.

### Phylogenetic analyses

Phylogenetic analyses were performed by searching homologous sequence data of the family Diatrypaceae in the GenBank database, selected from NCBI and recently published papers (Mehrabi et al. 2019; Dayarathne et al. 2020; Konta et al. 2020; Dissanayake et al. 2021; Zhu et al. 2021). After the preliminary identification results of the sequences, multiple sequence alignments (ITS and β-tubulin) were aligned using BioEdit v. 7.0 (Hall 1999). Alignments were converted from FASTA to PHYLIP format by using Alignment Transformation Environment online (https://sing.ei.uvigo.es/ALTER/, Glez-Peña et al. 2010). Maximum Likelihood (ML) analyses and Bayesian posterior probabilities (BYPP) were performed by using RAxML-HPC BlackBox (8.2.12) and MrBayes on XSEDE (3.2.7a) tools in the CIPRES Science Gateway platform, based on a combination of ITS and TUB2 sequence data (Miller et al. 2010). Both of the two methods use the GTR+I+G model of evolution (Nylander 2004). The Bootstrap supports of ML analyses were obtained by running 1,000 pseudo-replicates and BYPP using a simulation technique called Markov chain Monte Carlo (or MCMC) to approximate the posterior probabilities of trees. Six simultaneous Markov Chains were run for 3,000,000 generations and trees were sampled every 1,000^th^ generation. Finally, the tree was visualised in FigTree v.1.4.4 (Rambaut 2012) and edited by using Adobe Photoshop CS6 software. The final alignment and phylogenetic trees were deposited in TreeBASE under the submission ID28176 (http://www.treebase.org/)

## Result

### Phylogenetic analyses

**Figure 1.**
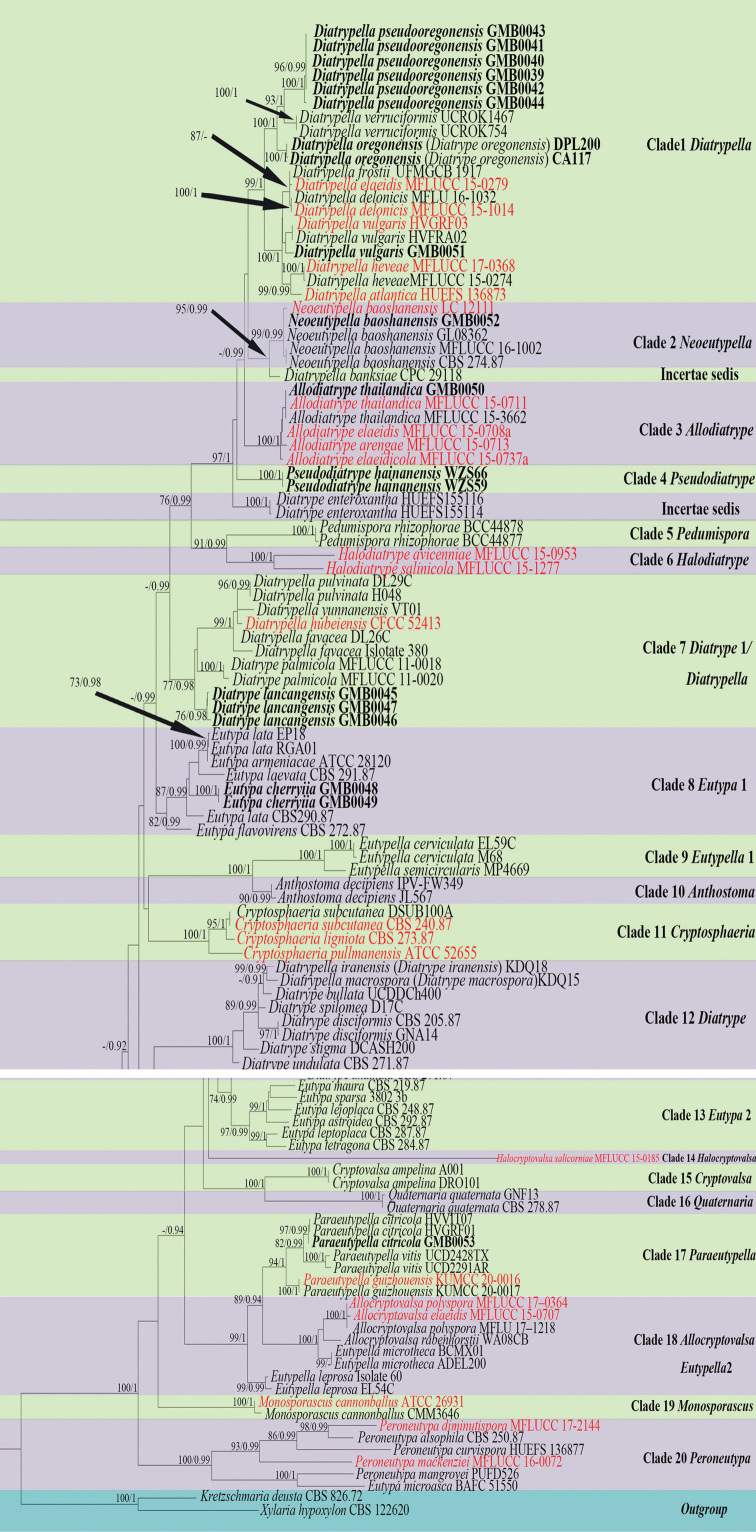
Phylogram generated from Maximum Likelihood (RAxML) analyses, based on ITS-β-tubulin matrix. ML bootstrap supports (≥ 70%) and Bayesian posterior probability (≥ 0.90) are indicated as ML/BYPP. The tree is rooted to *Kretzschmariadeusta* (CBS 826.72) and *Xylariahypoxylon* (CBS 122620). Ex-type strains are in red. Newly generated strains are in black bold.

Based on RAxML and BYPP analyses, phylogenetic analyses were similar in overall tree topologies and did not differ significantly. The dataset consists of 105 taxa for representative strains of species in Diatrypaceae, including outgroup taxa with 1071 characters, including gaps (ITS: 1–486, β-tubulin: 486–1071). The RAxML analyses resulted in a best scoring likelihood tree selected with a final ML optimisation likelihood value of -15731.506304, which is shown in Fig. 1.

The phylogenetic tree, based on combining ITS and β-tubulin sequence data, is also shown in Fig. 1 and contains 17 clades within Diatrypaceae. Below, we list the placements of new taxa:

Clade 1: *Diatrypellapseudooregonensis* and *Diatrypellaoregonensis* clustered with the species of *Diatrypella* in Clade 1 with high bootstrap support, *Diatrypellapseudooregonensis* is introduced as an 8-spored new species of *Diatrypella* and *Diatrypeoregonensis* is renamed as *Diatrypellaoregonensis*.

Clade 4: *Pseudodiatrype* formed a separate branch in a clade (Clade 4) basal to the genus *Allodiatrype*.

Clade 7: *Diatrypelancangensis* clusters with the species of *Diatrypella* and *Diatrype* in an unresolved clade. However, *Diatrype* and *Diatrypella* have previously shown confused classification which is difficult to distinguish, based on phylogenetic aspects alone. Therefore, we introduce *Diatrypelancangensis* as a new species of *Diatrype*, based on phylogenetic analyses and morphological differences (Table 2).

**Table 2.** The dimensions of the present species and some related species of *Diatrype* and *Allodiatrype*.

Species name	Stromata			Asci			Ascospores		Reference
	Length (mm)	Wide (mm)	Length (μm)		Wide (μm)	Length (μm)		Wide (μm)	
* Allodiatrype arengae *	0.69–0.94	0.37–0.93	54–109		6–10	7–10		2–3	Konta et al. (2020)
* A. elaeidicola *	1.2–2.8	0.9–1.66	60–91		4–7	8–10		1.5–3	Konta et al. (2020)
* A. elaeidis *	0.47–0.86	0.44–0.71	56–95		9–11	8–10		1.5–3	Konta et al. (2020)
* A. thailandica *	NA	1–2	55–80		5–7	3.8–6.9		1–1.4	Li et al. (2016)
* Diatrype acericola *	1–2	1–1.5	23–27		5–7	7.5–9		0.9–1.1	Vasilyeva and Ma (2014)
* D. albopruinosa *	0.5–1 diam.	0.5–1 diam	40–60		10–15	12–15		3.5–4	Vasilyeva and Ma (2014)
* D. bullata *	2–7 diam.	2–7 diam	25–30		5–7	7.5–9		Very thin	Vasilyeva and Ma (2014)
* D. disciformis *	NA	NA	75–115		NA	5–9		1.5–2	Senanayake et al. (2015)
* D. enteroxantha *	NA	1–3.5	18–28.5		5–9	7–10		1.5–2.5	de Almeida et al. (2016)
* D. hypoxyloides *	NA	NA	20–25		4–6	4–6		Very thin	Vasilyeva and Ma (2014)
*** D. lancangensis ***	**NA**	**NA**	**90.5–160.5**		**7–15**	**11–18.5**		**2–4**	**This study**
* D. lijiangensis *	1 diam.	1 diam	50–90		6–9	6–8		1–2	Thiyagaraja et al. (2019)
* D. macounii *	1–1.8 diam.	1–1.8 diam	25–30		4–6	4–6		0.7–1	Vasilyeva and Ma (2014)
* D. stigma *	NA	NA	25–30		5–7	6–8		1.5–2	Vasilyeva and Ma (2014)
* D. subundulata *	NA	NA	35–40		5–7	7–9		1.7–1.9	Vasilyeva and Ma (2014)
* D. undulata *	NA	NA	25–30		3.5–4.5	5–7		0.9–1.3	Vasilyeva and Ma (2014)
* D. whitmanensis *	NA	NA	50–82		8–15	7.5–10		1–1.5	Trouilla et al. 2010
*** Pseudodiatrype hainanensis ***	**2–3.6**	**1.6–3**	**110–155.5**		**6–10**	**8.5–13**		**1.5–2.5**	**This study**

Newly identified taxa are indicated in bold, NA: No description available.

Clade 8: *Eutypacerasi* forms a distinct lineage which is sister to *Eutypalata* (EP18, RGA01) (Fig. 1).

### Taxonomy

#### Diatrype

Taxon classificationFungiXylarialesDiatrypaceae

Fr.

0276BB73-FA2F-5A67-B8EC-6C93173AFA95

##### Notes.

The genus *Diatrype* was introduced by Fries (1849). The genus is characterised by stromata widely effuse or verrucose, flat or slightly convex, with discoid or sulcate ostioles at the surface, 8-spored and long-stalked asci and hyaline or brownish, allantoid ascospores. In this study, we introduce a new species of *Diatrype* from China.

#### Diatrype lancangensis

Taxon classificationFungiXylarialesDiatrypaceae

S.H. Long & Q. R. Li sp. nov.

8288B30C-73B3-5E39-B2E2-ACFC8A620A19

839655

Fig. 2

##### Holotype.

GMB0045.

##### Etymology.

Refers to the name of the location, where the type specimen was collected.

##### Description.

Saprobic on decaying branches of an unidentified plant. **Sexual morph**: *Stromata* immersed in bark, aggregated, irregular in shape, widely effused, flat, margin diffuse, surface dark brown to black, with punctiform ostioles scattered at surface, with tissues soft, white between perithecia. *Entostroma* dark with embedded perithecia in one layer. *Perithecium* semi-immersed in stroma, globose to subglobose, glabrous, with cylindrical neck, brevicollous or longicollous 283.5–343.5 μm high, 207–290 μm broad (av. = 315.5 × 248.0 μm, n = 10), ovoid, obovoid to oblong, monostichous, aterrimus. *Ostiole* opening separately, papillate or apapillate, central. *Peridium* 30–50 μm thick, dark brown to hyaline with *textura angularis* cell layers. *Asci* 90.5–160.5 × 7.0–15.0 μm (av. = 129.5 × 10.5 μm n = 30) 8-spored clavate, unitunicate, with rounded apex, apical rings inamyloid. *Ascospores* 11–18.5 × 2–4 μm (av. = 14.9 × 2.8 μm, n = 30), irregularly arranged, allantoid, slightly curved, brown to dark brown, smooth, aseptate, usually with oil droplets. Asexual morph: undetermined.

**Figure 2.**
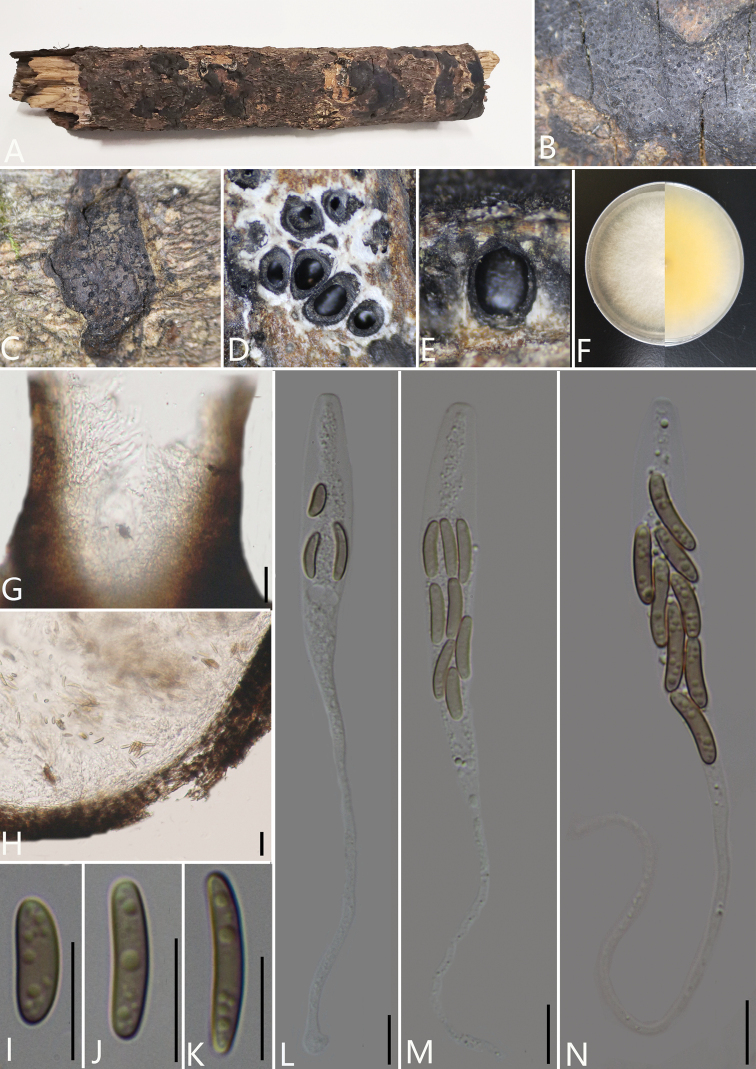
*Diatrypelancangensis* (GMB0045, **holotype**) **A** stromata on host substrate **B, C** stromata on host **D** transverse sections through ascostroma **E** vertical section through ascostroma **F** culture on PDA**G** ostiolar canal **H** peridium **I–K** ascospores **L–N** asci. Scale bars: 10 μm (**G–N**).

##### Culture characteristics.

Ascospores germinating on PDA within 24 hours. Colonies on PDA, white when young, became luteous, dense but, thinning towards edge, margin rough, white from above, reverse white at margin, pale yellow to luteous at centre, no pigmentation produced on PDA medium, no conidia observed on PDA or on OA media.

##### Specimens examined.

China, Yunnan Province, Baoshan City, Lancang River Nature Reserve (25°1'17.44"N, 99°35'10.05"E) on branches of an unidentified plant, 4 October 2019. Altitude: 2549 m., Y.H. Pi & Qiong Zhang, LC172 (GMB0045, ***holotype***, KUN-HKAS 112664, ***isotype***, ex-type living culture GMBC0045).

##### Additional specimens examined.

China, Yunnan Province, Baoshan City, Lancang River Nature Reserve (25°1'17.44"N, 99°35'10.05"E) on branches of an unidentified plant, 4 October 2019. Altitude: 2549 m., Y.H. Pi and Qiong Zhang, LC173 (GMB0046, KUN-HKAS 112665, living culture GMBC0046); CHINA, Yunnan Province, Baoshan City, Lancang River Nature Reserve (25°1'15.48"N, 99°35'24.08"E) on branches of an unidentified plant, 5 October 2019. Altitude: 2623 m., Y.H. Pi and Qiong Zhang, LC262 (GMB0047, KUN-HKAS 112672, living culture GMBC0047).

##### Additional sequences.

GMB0045 (LSU: MW797057, RPB2: MW81490); GMB00046 (LSU: MW797058); GMB0047 (LSU: MW797060, RPB2: MW814903)

##### Note.

Our new strain, GMBC0045 falls into the unresolved clade (Clade 7) which comprises five *Diatrypella* and one *Diatrype* species (Fig. 1), this clade is consistent with the study of Konta et al. (2020). The taxonomic confusion of Diatrypaceae has led to difficulties in separating the genera. We consider that the new species belongs to the genus *Diatrype*, based on the stromata features mentioned above which closely resemble descriptions of *Diatrypesubundulata* Lar. N. Vassiljeva & Hai X. Ma and *Diatrypeundulata* (Pers.) Fr. (Vasilyeva et al. 2014). However, the ascospores of these species are larger than the ascospores of *D.subundulata* and *D.undulata* (Table 2). Phylogenetic analyses also showed that *D.lancangensis* falls on a separate branch that clustered with species of *Diatrypella* and *Diatrype* (Fig. 1). Hence, by combining morphological characteristics and phylogenetic analyses, it seems appropriate to categorise this species as *Diatrype*.

In the phylogenetic analyses, it can be seen that Clade 7 can be defined as a new genus, but it is difficult to find the common morphological similarities among these species. More specimens and sequence or chemical composition analysis are needed in the future to determine whether Clade 7 can be a new genus. The characteristics of the stromata of *Diatrypella* spp. in clade 7 are solitary and scattered, which is distinctly different from widely effuse, flat and slightly convex stromata of *Diatrypelancangensis* and *Diatrypepalmicola* (Liu et al. 2015; Hyde et al. 2020b; Zhu et al. 2021). And in the recent study, Zhu et al. (2021) proposed that the species of *Diatrypella* in Clade 7 were isolated from *Betula* spp., it may have host specificity. Because of the above two reasons, we think it is better to classify our strains into *Diatrype*.

#### Pseudodiatrype

Taxon classificationFungiXylarialesDiatrypaceae

S.H. Long & Q.R. Li gen. nov.

0A1D8CCD-7E56-5FB2-B935-86DBE143A32F

839658

##### Etymology.

Refers to this genus resembling *Diatrype* in morphology, but it is phylogenetically distinct.

##### Type species.

*Pseudodiatrypehainanensis* S. H. Long & Q.R. Li sp. nov.

##### Description.

*Saprobic* on decaying branches of an unidentified plant. **Sexual morph**: *Stromata* scattered or aggregated on host, wart-like, pustulate, visible as black, rounded to irregular in shape on host surface, erumpent through host bark, 5–20 ascomata immersed in one stroma. *Endostroma* consists of outer layer of black, small, dense, thin parenchymal cells and inner layer of white, large, loose parenchymal cells, thin, pale yellow, powdery near margin of the black cells. *Ostiole* opening through host bark and appearing as black spots, separately, papillate or apapillate, central. *Perithecium* immersed in stroma, globose to subglobose, glabrous, with cylindrical neck, brevicollous or longicollous. *Peridium* is composed of an outer layer of dark brown to black, thin-walled cells, arranged in *textura angularis*, the inner layer of hyaline thin-walled cells of *textura angularis*. *Asci* 8-spored, unitunicate, clavate, long-stalked, apically rounded, apical rings inamyloid. *Ascospores* irregularly arranged, allantoid, slightly or moderately curved, smooth, subhyaline, aseptate, usually with two oil droplets. **Asexual morph**: undetermined.

##### Note.

The genus *Pseudodiatrype* is introduced to accommodate the new collection made from Hainan Province of China and typified by *Pseudodiatrypehainanensis*. *Pseudodiatrype* is monotypic and, morphologically, resembles *Diatrype* and *Allodiatrype* Konta & K.D. Hyde. However, *Pseudodiatrype* can be distinguished from *Diatrype* by its 5–20 ascomata immersed in a stroma, while the stroma of species of *Diatrype* is distributed over large areas, sometimes covering the surface of the host (Vasilyeva and Ma 2014; Konta et al. 2020). *Pseudodiatrype* differs from *Alloiatrype* by having its 5–20 ascomata immersed in a stroma, whereas the stroma of *Allodiatrype* has only 1–10 ascomata. Moreover, the endostroma of *Allodiatrype* is composed of dark brown outer layer cells and yellow inner layer cells (Konta et al. 2020), which are different from the endostroma of *Pseudodiatrype* having black outer and inner cells surrounded by powdery, pale yellow cells. In addition, the sizes of stroma and ascospores are different from species of *Diatrype* and *Allodiatrype* (Table 2). In the phylogenetic analyses, species of *Pseudodiatrype* appeared in a separate branch which is distinct from other genera within *Diatrypaceae* (Fig. 1), thus, justifying the erection of the new genus *Pseudodiatrype*.

#### Pseudodiatrype hainanensis

Taxon classificationFungiXylarialesDiatrypaceae

S. H. Long & Q.R. Li sp. nov.

6A6C31F6-E423-5CAF-AF06-EF1DD9BAF9E4

839659

Fig. 3

##### Holotype.

GMB0054.

##### Etymology.

Refers to the location of collections, Hainan Province.

##### Description.

*Saprobic* on decaying branches of an unidentified plant. **Sexual morph**: *Stromata* wart-like, pustulate, 2–3.6 mm long and 1.6–3 mm broad (av. = 3.2 × 1.9 mm, n = 30), about 2 mm thick, 5–20 in single stroma, visible as black, rounded to irregular in shape on the host surface, erumpent through host bark, solitary to gregarious. *Endostroma* composed of an outer layer of dark brown to black, small, tightly packed, thin parenchymatous cells and an inner layer of white, large, loose parenchymal cells with powdery, thin, yellowish tissue. *Ostiole* opening separately, papillate or apapillate, central. *Perithecium* immersed in the stroma, globose to subglobose, glabrous, with cylindrical neck, brevicollous or longicollous, 193–347 μm high, 138–206 μm diam. (av. = 278 × 156 μm, n = 10). *Peridium* 30–50 μm thick, dark brown to hyaline with *textura angularis* cell layers. *Asci* 110–155.5 × 6–10 μm (av. = 132 × 8 μm, n = 30), 8-spored, unitunicate, clavate, long-stalked, apically rounded with inamyloid rings. *Ascospores* 8.5–13 × 1.5–2.5 μm (av. = 10.5 × 2 μm, n = 30), irregularly arranged, allantoid, slightly or moderately curved, smooth, subhyaline, aseptate, usually with two oil droplets. **Asexual morph**: undetermined.

**Figure 3.**
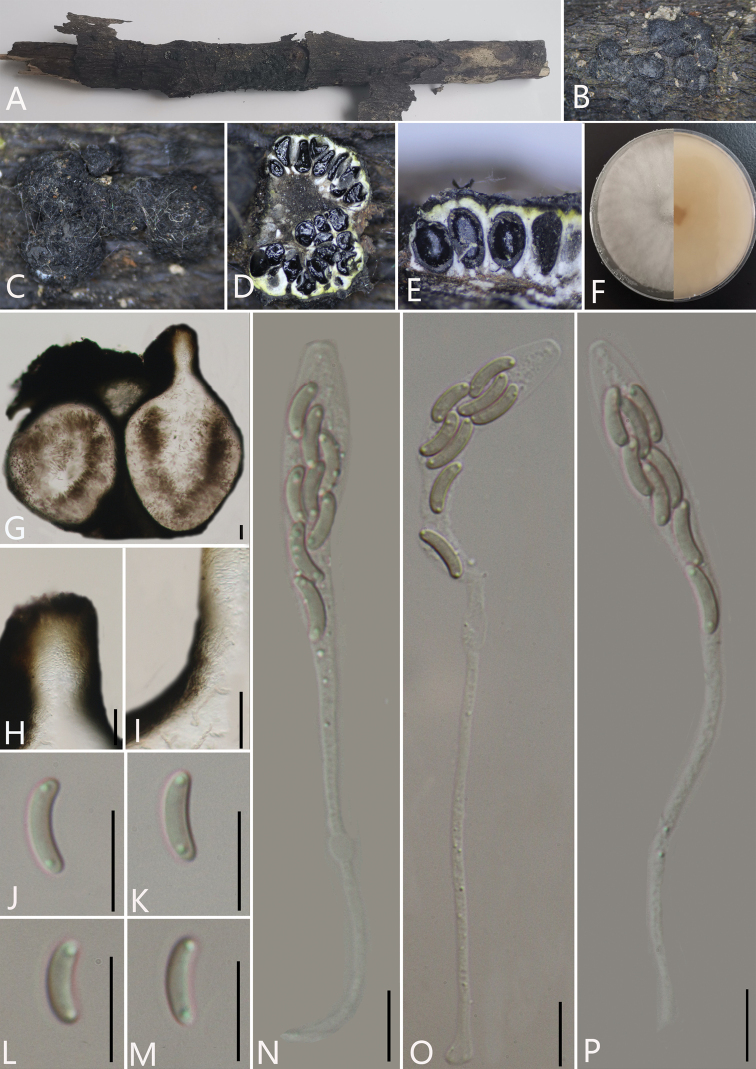
*Pseudodiatrypehainanensis* (GMB0054, **holotype**) **A** stromata on host substrate **B, C** stromata on host **D** transverse section through ascostroma **E** vertical section through ascostroma **F** culture on PDA**G** section through the ascostroma **H** ostiolar canal **I** peridium **J–M** ascospores **N–P** asci. Scale bars: 40 μm (**G**); 10 μm (**H–P**).

##### Culture characteristics.

Ascospores germinating on PDA within 24 hours. Colonies on PDA, white when young, became pale brown, dense, but thinning towards edge, fluffy to slightly fluffy, white from above, pale brown from below, no pigmentation produced on PDA medium, no conidia observed on PDAor on OA media.

##### Specimens examined.

China, Hainan Province, Wuzhishan City, Wuzhishan Nature Reserve (18°54'21.81"N, 109°40'54.12"E) on branches of unidentified plant, 14 November 2020. Altitude: 775 m. Y.H. Pi & Q.R. Li, WZS59 (GMB0054, ***holotype***, KUN-HKAS 112700, ***isotype***, ex-type living culture GMBC0054).

##### Additional specimen examined.

China, Hainan Province, Wuzhishan City, Wuzhishan Nature Reserve (18°54'21.81"N, 109°40'54.12"E) on branches of an unidentified plant, 14 November 2020. Altitude: 775 m, Y.H. Pi & Q.R. Li, WZS66 (GMB0055, living culture GMBC0055)

##### Additional sequences.

GMB0054 (LSU: MW797055, RPB2: MW814900); GMB0055 (LSU: MW797056, RPB2 MW814901).

##### Note.

A peculiar feature of *Pseudodiatrypehainanensis* is the composition of endostroma. There are black outer layer cells, white inner layer cells and powdery, yellowish cells that are smaller than the white cells at the edge of the endostroma near the black cells in endostroma.

#### Diatrypella

Taxon classificationFungiXylarialesDiatrypaceae

(Ces. & De Not.) De Not.

F0FBFC39-56D6-50C4-84C9-387206A5D730

##### Notes.

The genus *Diatrypella* was introduced by Cesati & De Notaris (1863) and was typified with *Diatrypellaverruciformis* (Ehrh.) Nitschke. This genus was characterized by pustule-like stromata erumpent through the host surface, polysporous asci and allantoid ascospores and libertella-like asexual morphs (Senanayake et al. 2015; Hyde et al. 2017; Shang et al. 2017). In this study, we introduce a new species, a new combination and a new record of *Diatrypellavulgaris* from Guizhou Province for China.

#### Diatrypella pseudooregonensis

Taxon classificationFungiXylarialesDiatrypaceae

S.H. Long & Q.R. Li sp. nov.

37571EF4-7139-58DD-BEC9-9D90BF0DB8D7

839656

Fig. 4

##### Holotype.

GMB0041

##### Etymology.

Refers to its similar species of *Diatrypeoregonensis*.

##### Description.

*Saprobic* on decaying branches of unidentified plant. **Sexual morph**: *Stromata* pustulate, with groups of 3–16 perithecia, rugose, visible as black, erumpent, scattered, surrounded by a thin, black line in host tissue, solitary to gregarious, 1–3 mm long and 0.5–2 mm broad (av. = 2 × 1.5 mm, n = 30), about 1 mm thick. *Endostroma* white to light yellow. *Ostiole* opening separately, papillate or apapillate, central. *Perithecium* immersed in stroma, globose to subglobose, glabrous, with cylindrical neck, brevicollous or longicollous 218.5–465 μm high, 112–257 μm diam. (av. = 306 × 164 μm, n = 10), globose to subglobose, glabrous, ostioles individual. *Peridium*: 30–50 μm thick, dark brown to hyaline with *textura angularis* cell layers. *Asci* 95–149 × 6.5–11.5 μm (av. = 120 × 10.5 μm, n = 30), 8-spored, unitunicate, clavate or cylindrical, long-stalked, apically rounded, apical rings inamyloid. *Ascospores* 11–16 × 1.5–3.5 μm (av. = 14 × 2.5 μm, n = 30), irregularly arranged, allantoid, slightly or moderately curved, subhyaline to slightly brown, smooth, aseptate, usually with two oil droplets. **Asexual morph**: undetermined.

**Figure 4.**
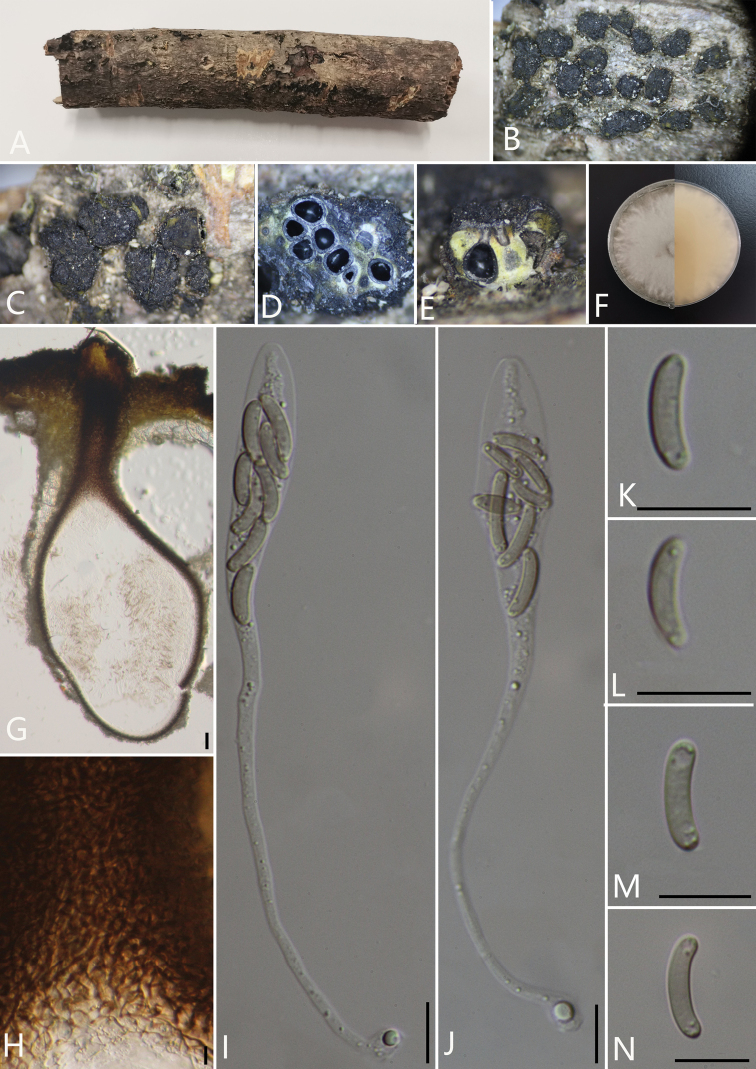
*Diatrypellapseudooregonensis* (GMB0041, **holotype**) **A** stromata on host substrate **B, C** stromata on host substrate **D** transverse section through ascostroma **E** vertical section through ascostroma **F** culture on PDA**G** section through the ascostroma **H** ostiolar canal **I, J** asci **K–N** ascospores. Scale bars: 20 μm (**G**); 10 μm (**H–N**).

##### Culture characteristics.

Ascospores germinating on PDA within 24 hours. Colonies on PDA, white when young, became pale brown, dense, but thinning towards the edge, margin rough, white from above, white at margin and light brown at centre from below, no pigmentation produced on PDA medium, no conidia observed on PDA or on OA media.

##### Specimens examined.

China, Yunnan Province, Baoshan City, Lancang River Nature Reserve (25°1'19.88"N, 99°35'30.68"E) on branches of an unidentified plant, 5 October 2019. Altitude: 2677 m, Y.H. Pi & Qiong Zhang, LC323 (GMB0041, ***holotype***, KUN-HKAS 112646, ***isotype***, ex-type living culture GMBC0041)

##### Additional specimens examined.

China, Yunnan Province, Baoshan City, Lancang River Nature Reserve (25°1'13.51"N, 99°35'25.59"E) on branches of an unidentified plant, 6 October 2019. Altitude: 2630 m, Y.H. Pi & Qiong Zhang, LC384 (GMB0043, KUN-HKAS 112681, living culture GMBC0043); China, Yunnan Province, Baoshan City, Lancang River Nature Reserve (25°1'15.00"N, 99°35'39.73"E) on branches of an unidentified plant, 5 October 2019. Altitude: 2698 m, Y.H. Pi & Qiong Zhang, LC312 (GMB0040, KUN-HKAS 112674, living culture GMBC0040); China, Yunnan Province, Baoshan City, Lancang River Nature Reserve (25°35'19.09"N, 99°35'19.09"E) on branches of an unidentified plant, 5 October 2019. Altitude: 2569 m, Y.H. Pi & Qiong Zhang, LC193 (GMB0039, KUN-HKAS 112667, living culture GMBC0039); China, Yunnan Province, Baoshan City, Lancang River Nature Reserve (25°1'9.11"N, 99°35'24.80"E) on branches of an unidentified plant, 5 October 2019. Altitude: 2649 m, Y.H. Pi & Qiong Zhang, LC335 (GMB0042, KUN-HKAS 112647, living culture GMBC0042); China, Guizhou Province, Anshun City, Pingba District (26°25'9.65"N, 106°24'24.48"E) on branches of an unidentified plant, 1 August 2020. Altitude: 1250 m, Y.H.Pi, PB51 (GMB0044, KUN-HKAS 112693, living culture GMBC0044).

**Additional sequences.** GMB0041 (LSU: MW797062, RPB2: MW814906); GMB0043 (LSU: MW797064, RPB2: MW814907); GMB0040 (LSU: MW797061, RPB2: MW814905); GMB0039 (LSU: MW797059, RPB2: MW814904); GMB0042 (LSU: MW797063); GMLB0044 (LSU: MW979054, RPB2: MW814899).

**Note.** Morphologically, *Diatrype* has 8 ascospores in a single ascus, while *Diatrypella* has more than eight ascospores in each ascus (Senanayake et al. 2015). However, previous research (e.g. Acero et al. 2004 and Trouillas et al. 2011) suggested that both *Diatrypella* and *Diatrype* are polyphyletic within the family. In the phylogenetic analyses, *Diatrypellapseudooregonensis* grouped closely to the *D.verruciformis* and thus, we consider this new species to belong in the genus *Diatrypella*, because it is doubtful whether the number of ascospores per asci is useful as a basis for generic classification.

#### Diatrypella vulgaris

Taxon classificationFungiXylarialesDiatrypaceae

Trouillas, W.M. Pitt & Gubler, Fungal Diversity 49: 212 (2011)

6D81ECFA-42D3-5AD0-9A74-63C6F55ADEEE

519404

Fig. 5

##### Description.

*Saprobic* on decaying branches of an unidentified plant. **Sexual morph**: *Stromata* scattered on the host, 0.8–1.5 mm long and 0.8–2 mm broad (av. = 1.2 × 1.3 mm, n = 30) pustulate, visible as black, rounded to irregular in shape on host surface, semi-immersed, erumpent through host bark, with 2–8 ascomata immersed in one stroma. *Endostroma* consists of outer dark brown, small, dense, thin parenchymal cells and an inner layer of white, large, loose parenchymal cells. *Ostiole* opening separately, papillate or apapillate, central 710.7–787.2 μm high, 270.2–422 μm diam. (av. = 742 × 363 μm, n = 10). *Perithecium* immersed in stroma, round to oblong, with cylindrical neck, brevicollous or longicollous. *Peridium* composed of outer layer of dark brown to black, thin-walled cells, arranged in *textura angularis*, inner layer of hyaline thin-walled cells of *textura angularis*. *Asci* 111.4–152.9 × 10.6–17.5 μm (av. = 124.5 × 15.5 μm, n = 30), polysporous, clavate, long-stalked, apically rounded. *Ascospores* 8–11 × 1–2 μm (av. = 8.9 × 1.7 μm, n = 30), overlapping, crowded, allantoid, slightly or moderately curved, smooth, subhyaline, yellowish in mass, aseptate, usually with two oil droplets. **Asexual morph**: undetermined.

**Figure 5.**
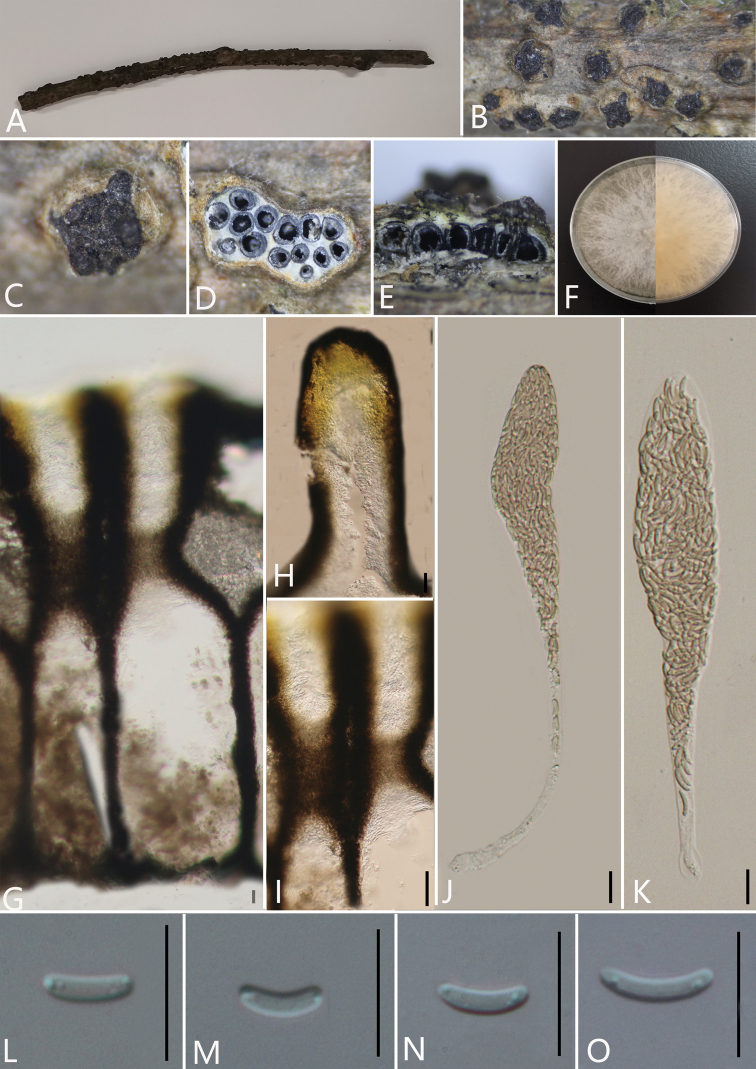
*Diatrypellavulgaris* (GMB0051, **new record for China**) **A** stromata on host substrate; **B, C** close-up of stroma **D** transverse sections through ascostroma **E** vertical section through ascostroma **F** culture on PDA**G** section through the ascostroma **H, I** ostiolar canal **J, K** asci **L–O** ascospores. Scale bars: 20 μm (**G**); 10 μm (**H–I**).

##### Culture characteristics.

Ascospores germinating on PDA within 24 hours. Colonies on PDA, white when young, became pale brown, dense, but thinning towards edge, medium dense, white from above, reverse side white at margin, flesh to pale brown at centre, no pigmentation produced on PDA medium, no conidia observed on PDA or on OA media.

##### Specimens examined.

China, Guizhou Province, Guiyang City, Gaopo Township (26°29'72.02"N, 106°29'55.57"E), on branches of unidentified plant, 30 October 2020. Altitude: 1589 m, S.H. Long, GP02 (GMB0051, KUN-HKAS 112697, living culture GMBC0051).

##### Additional sequences.

GMB0051 (LSU: MW797051, RPB2: MW814897).

##### Note.

The comparison of ITS sequences in NCBI showed that this isolate is 100% similar to the strain of *Diatrypellavulgaris* (HVGRF03), isolated from holotype specimens. Morphologically, GMB0051 shows the same features as *Diatrypellavulgaris*. The stromata of these specimens are similar, but ascospores of GMB0051 are thinner than those of the HVGRF03 (8–10 × 2–2.5 μm) and, when compared with the ascospores of strain MFLUCC 17-0128 (4.5–7.5 × 1–2 μm), they are shorter than GMB0051 (Trouillas et al. 2011; Hyde et al. 2017). Here, we use the ITS sequence similarity between the new collection and the type strain of *Diatrypellavulgaris* as the identification tool. *Diatrypellavulgaris* has been reported in Austria and Thailand (Trouillas et al. 2011, Hyde et al. 2017). This is the first report of *Diatrypellavulgaris* from China.

#### Diatrypella oregonensis

Taxon classificationFungiXylarialesDiatrypaceae

(Wehm.) S.H. Long & Q.R. Li comb. nov.

7CE88264-5FFE-5E09-8396-D563E25E6945

839728

≡ Eutypellaoregonensis Wehm. Pap. Mich. Acad. Sci. 11: 163 (1930)
≡ Diatrypeoregonensis (Wehm.) Rappaz, Mycol. helv. 2(3): 420 (1987)

##### Description.

See Trouillas et al. (2010).

##### Note.

The strains of *Diatrypeoregonensis* (DPL200, CA117) generated from Trouillas et al. (2010) grouped in *Diatrypella s. str. Diatrypeoregonensis* was erected in 1930 as *Eutypellaoregonensis* (Kauffman 1930). No available sequences from type material were found. After re-examination of holotype specimen of *Diatrypeoregonensis*, Trouillas et al. (2010) introduced two strains of *Diatrypeoregonensis* (DPL200 and CA117). Although neither of these strains are ex-type, they are, the most authoritative strains. Here, we tentatively transfer *Diatrypeoregonensis* to *Diatrypella* as *Diatrypellaoregonensis*, based on the phylogenetic analyses (Fig. 1). *Diatrypellaoregonensis* is similar to *D.pseudooregonensis* in having 8-spored asci (Rappaz 1987; Trouillas et al. 2011). Nevertheless, we consider that the number of ascospores as a basis for distinguishing *Diatrypella* from *Diatrype* is not useful.

#### Allodiatrype

Taxon classificationFungiXylarialesDiatrypaceae

Konta & K.D. Hyde Mycosphere 11(1): 247 (2020)

2D755444-3424-5C34-8896-0131868E17B4

##### Notes.

The genus *Allodiatrype* was introduced by Konta et al. (2020), which was characterised by regular or irregular-shaped stromata, erumpent through host surface, asci with 8 spores and aseptate, allantoid ascospores. In this study, we introduce a new record of *Allodiatrypethailandica* (R.H. Perera et al.) Konta & K.D. Hyde collected from Yunnan Province in China.

#### Allodiatrype thailandica

Taxon classificationFungiXylarialesDiatrypaceae

(R.H. Perera et al.) Konta & K.D. Hyde, Mycosphere 11(1): 253 (2020)

36962663-357D-564A-B748-708C9118D11B

556932

Fig. 6

≡ Diatrypethailandica R.H. Pereraet al., Fungal Diversity 78: 1–237, [105] (2016)

##### Description.

*Saprobic* on decaying branches of unidentified plant. **Sexual morph**: *Stromata* wart-like, pustulate, 0.5–1.8 mm long and 0.8–2.2 mm broad (av. = 1.2 × 1.3 mm, n = 30), about 1 mm thick, 1–18 in a single stroma, visible as black, rounded to irregular in shape on the host surface, erumpent through host bark, solitary to gregarious. *Endostroma* composed of an outer layer of dark brown to black, small, tightly packed, thin parenchymatous cells and an inner layer of white to yellow, large, loose parenchymal cells. *Ostiole* opening separately, papillate or apapillate, central. *Perithecium* immersed in stroma, globose to subglobose, glabrous, with cylindrical short neck, 377–447 μm high, 191–264 μm diam. (av. = 406 × 221 μm, n = 10). *Peridium* hyaline to dark brown with *textura angularis* cell layers. *Asci* 80–113.5 × 6.9–10 μm (av. = 109.3 × 8.5 μm, n = 30), 8-spored, unitunicate, clavate, long-stalked, upper part inflated, apically rounded to truncate, apical rings inamyloid. *Ascospores* 6–11 × 2–2.5 μm (av. = 8.9× 2.3 μm, n = 30), irregularly arranged, allantoid, slightly curved, smooth, subhyaline, aseptate, usually with two oil droplets. **Asexual morph**: undetermined.

**Figure 6.**
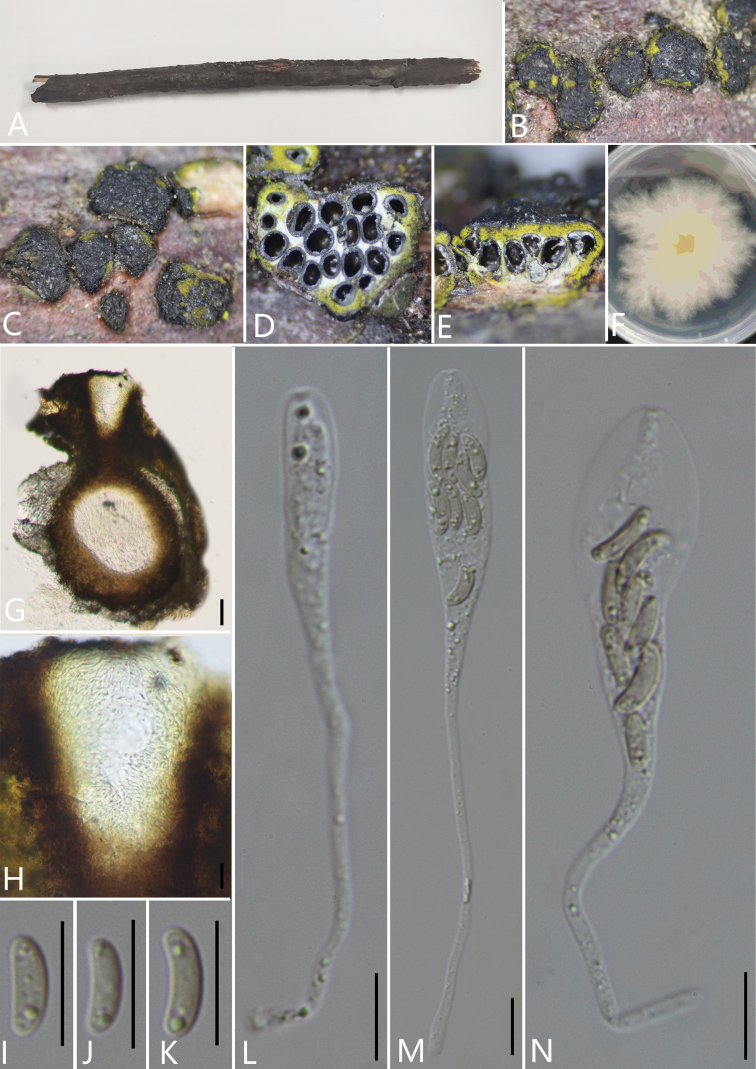
*Allodiatrypethailandica* (GMB0050, new record for China) **A** stromata on host substrate **B, C** close-up of stromata **D** transverse section through ascostroma **E** vertical section through ascostroma **F** culture on PDA**G** section through the ascostroma **H** ostiolar canal **I–K** ascospores **L–N** asci. Scale bars: 20 μm (**G**); 10 μm (**H–N**).

##### Culture characteristics.

Ascospores germinating on PDA within 24 hours. Colonies on PDA, white when young, became pale yellow, irregular in shape, medium dense, flat or effuse, slightly raised, with edge fimbriate, fluffy to fairly fluffy, white from above, reverse side white at margin, pale brown at centre, no pigmentation produced on PDA medium, no conidia observed on PDA or on OA media.

##### Specimens examined.

China, Yunnan Province, Baoshan City, Lancang River Nature Reserve (24°57'25.35"N, 99°44'22.82"E), on branches of unidentified plant, 2 October 2019. Altitude: 1317 m, Y.H. Pi & Qiong. Zhang, LC103 (GMB0050, KUN-HKAS 112660, living culture GMBC0050).

##### Additional sequences.

GMB0050 (LSU: MW797052).

##### Note.

The ITS sequence data were subjected to BLAST in NCBI and the results showed that it is 100% similar to *Allodiatrypethailandica*. Additionally, based on morphological and phylogenetic analyses, this strain was identified as the *A.thailandica*. The stromata are similar, but the ascospores of GMB0050 are longer and wider than the ascospores of strain MFLUCC 15-3662 (3.8–6.9 × 1–1.4 μm) isolated from the holotype specimen, but it is similar to the strain MFLU 17-0735 (6.5–10.7 × 1.6–2.7 μm) (Perera et al. 2020). Here, we use the ITS sequence similarity between the new collection and the type strain of *Allodiatrypethailandica* as basis for identification. *A.thailandica* has been reported in Thailand in 2016 as *Diatrypethailandica* and recognised as *A.thailandica* by Konta et al. (2020). This is the first report of *Allodiatrypethailandica* from China.

#### Neoeutypella

Taxon classificationFungiXylarialesDiatrypaceae

M. Raza, Q.J. Shang, Phookamsak & L. Cai, Fungal Diversity 95: 167 (2019)

1FECB933-4C21-509B-B6C5-AF1A84D07C29

##### Note.

The genus *Neoeutypella* was introduced by Phookamsak et al. (2019) and is characterised by carbonaceous stromata immersed or semi-immersed on the host, 8-spored asci and hyaline or pale reddish-brown to brown ascospores. In this study, we introduce a new collection of *N.baoshanensis*, isolated from Guizhou Province in China.

#### Neoeutypella baoshanensis

Taxon classificationFungiXylarialesDiatrypaceae

M. Raza, Q.J. Shang, Phookamsak & L. Cai, Fungal Diversity 95: 168 (2019)

E313872E-9680-523B-9166-07E30D68E018

555372

Fig. 7

##### Description.

see Phookamsak et al. (2019).

##### Specimens examined.

China, Guizhou Province, Guiyang City, Gaopo Township (26°29'72.37"N, 106°29'59.33"E), on branches of unidentified plant, 30 November 2020. Altitude: 1589 m, S.H. Long, GP01 (GMB0052, KUN-HKAS 112696, living culture GMBC0052).

##### Additional sequences.

GMB0052 (LSU: MW797050, RPB2: MW814896).

##### Note.

The morphological characteristics of this specimen are consistent with those of *N.baoshanensis* a species described by Phookamsak et al. (2019). Based on phylogenetic and morphological analyses, we consider that this specimen is *Neoeutypellabaoshanensis*. *Neoeutypellabaoshanensis* was described as the type species of *Neoeutypella* on dead wood of *Pinusarmandii* Franch. from Yunnan Province in China (Phookamsak et al. 2019). This is the first record of *N.baoshanensis* from Guizhou Province, China.

**Figure 7.**
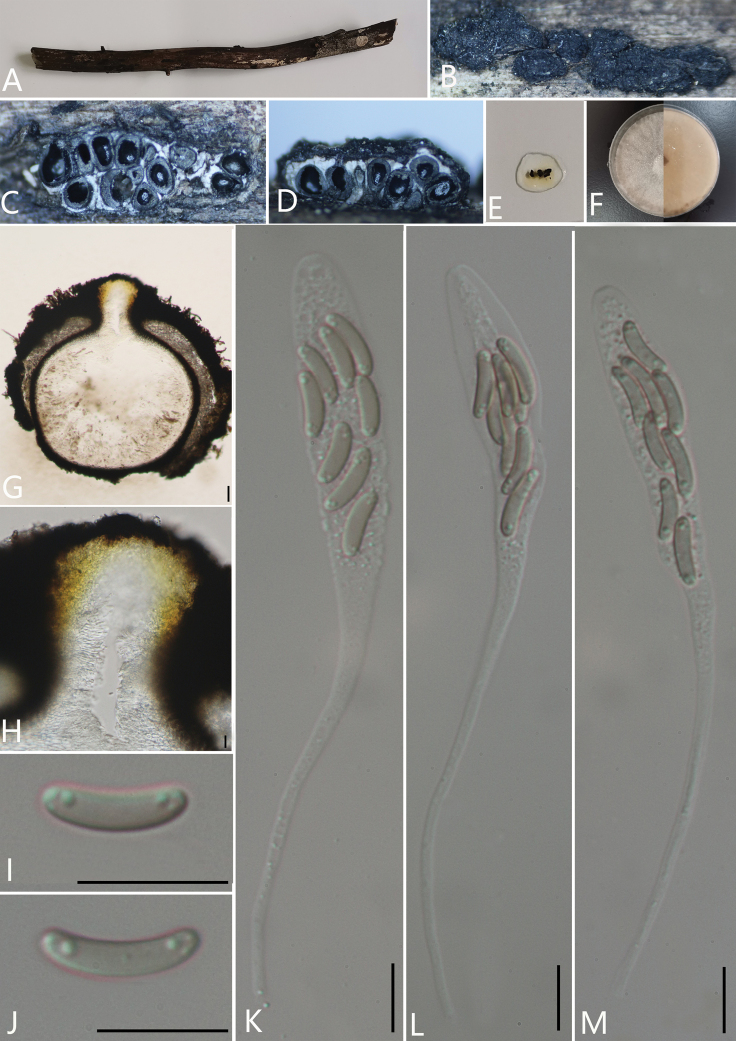
*Neoeutypellabaoshanensis* (GMB0052) **A** stromata on host substrate **B** close-up of stromata **C** transverse section through ascostroma **D** vertical section through ascostroma **E** pigments in KOH **F** culture on PDA**G** section through the ascostroma **H** ostiolar canal **I, J** ascospores **K–M** asci. Scale bars: 20 μm (**G**); 10 μm (**H–M**).

#### Eutypa

Taxon classificationFungiXylarialesDiatrypaceae

Tul. & C. Tul.

5A3A837E-F405-5164-BF91-558F6E8747AA

##### Notes.

Tulasne & Tulasne (1863) introduced the genus *Eutypa* with *Eutypalata* as the type species. This genus includes several phytopathogens, such as *E.lata* (Pers.) Tul. & C. Tul. and *E.leptoplaca* (Durieu & Mont.) Rappaz (Moyo et al. 2017). The morphological characteristics of this genus are black, rounded to irregular-shaped stromata on the host surface, erumpent through host epidermis, solitary to gregarious, entostromatic region, consisting of white pseudoparenchymatous cells and thin black pseudoparenchymatous tissue around the white entostroma, 8-spored, spindle-shaped asci and hyaline, oblong to allantoid ascospores (Rappaz 1987; Moyo et al. 2017). We introduce a new species of *Eutypa* collected from Guizhou Province in China.

#### Eutypa cerasi

Taxon classificationFungiXylarialesDiatrypaceae

S.H. Long & Q.R. Li sp. nov.

DB1D9A3E-0AD8-5603-92C9-D1503871C7BE

839657

Fig. 8

##### Holotype.

GMB0048.

##### Etymology.

Refers to its host, *Prunuscerasus*.

##### Description.

*Saprobic* on decaying branches of *Prunuscerasus*. **Sexual morph**: *Stromata* immersed in bark, covering surface of host, irregular in shape, widely effused, flat, margin diffuse, surface dark brown to black, with punctiform ostioles scattered at surface. Endostroma consists of an outer layer of black, small, dense, thin parenchymal cells and an inner layer of white, large, loose parenchymal cells. *Perithecium* semi-immersed in stroma, globose to subglobose, glabrous, with cylindrical neck, brevicollous 203–304 μm high, 346–477 μm diam. (av. = 408 × 250 μm, n = 10), ovoid, obovoid to oblong. *Ostiole* opening separately, papillate or apapillate, central. *Peridium* 30–50 μm thick, dark brown to hyaline with *textura angularis* cell layers. *Asci* 83.2–120 × 5.1–8.2 μm (av. = 104.4 × 6.3 μm n = 30) 8–spored clavate, unitunicate, rounded to truncate apex, apical rings inamyloid. *Ascospores* 7.3–9.9 × 1.4–2 μm (av. = 8.5 × 1.7 μm, n = 30), overlapping, allantoid, slightly curved, subhyaline, smooth, aseptate, usually with oil droplets. **Asexual morph**: undetermined.

**Figure 8.**
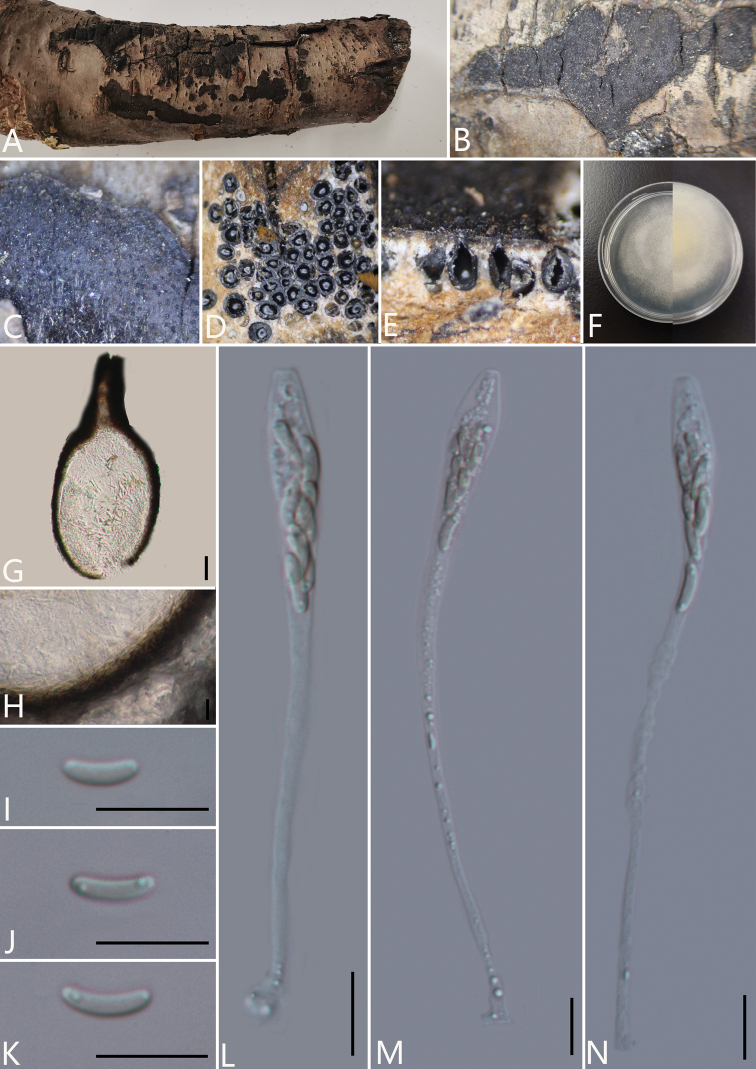
*Eutypacerasi* (GMB0048, **holotype**) **A** stromata on host substrate **B, C** close-up of stroma **D** transverse section through ascostroma **E** vertical section through ascostroma **F** culture on PDA**G** section through the ascostroma **H** peridium **I–K** ascospores **L–N** asci. Scale bars: 20 μm (**G**); 10 μm (**H–N**).

##### Culture characteristics.

Ascospores germinating on PDA within 24 hours. Colonies on PDA, white when young, became pale yellow, irregular in shape, medium dense, flat or effuse, white from above, reverse white at margin, pale yellow at centre, no pigmentation produced on PDA medium, no conidia observed on PDA or on OA media.

##### Specimens examined.

China, Guizhou Province, Guiyang City, Aha Lake National Wetland Park (26°32'50.21"N, 106°40'15.78"E), on branches of *Prunuscerasus*, 12 August 2020. Altitude: 1089 m, S.H. Long, AH4 (GMB0048, ***holotype***, KUN-HKAS 112685, ***isotype***, ex-type living culture GMBC0048).

##### Additional specimens examined.

China, Guizhou Province, Guiyang City, Aha Lake National Wetland Park (26°32'47.79"N, 106°40'21.09"E), on branches of *Cerasus* sp., 12 August 2020. Altitude: 1089 m, S.H. Long, AH40 (GMB0049, KUN-HKAS 112683, living culture GMBC0049).

##### Additional sequences.

GMB0048 (LSU: MW797048, RPB2: MW814894); GMB0049 (LSU: MW797049, RPB2: MW814895).

##### Notes.

*Eutypalata* is an important pathogen that has a wide range of hosts. However, the classification of *E.lata* is confusing because there are many variants in previous studies; now all are classified as *E.lata* (Index Fungorum 2020). Morphologically, the new collection GMB0048 has similar stromata with *Eutypalata*, but the ascomata of the new collection are smaller than the ascomata (400 μm diam.) of the original description of *E.lata* (Tulasne & Tulasne, 1863). The ascomata and asci of the new collection are smaller than the ascomata (400–600 μm diam.) and asci (110–180 × 5–7 μm) of the description of *E.lata* (Rappaz 1987). Additionally, in the phylogenetic analyses, *E.cerasi* is located on a branch that forms a sister clade with EP18 and RGA01 and CBS 290.87 basal to *E.cerasi*. Therefore, combining phylogenetic and morphological analyses, we introduce *Eutypacerasi* as a new species of *Eutypa*.

#### Paraeutypella

Taxon classificationFungiXylarialesDiatrypaceae

L.S. Dissan., J.C. Kang, Wijayaw. & K.D. Hyde.

60B185EE-60A6-5A22-8C90-05731A45D78E

##### Notes.

*Paraeutypella* was introduced by Dissanayake et al. (2021) to accommodate *Paraeutypellaguizhouensis* and the genus currently comprises three species. The genus is characterised by poorly developed stromata erumpent through the bark, grouped and irregularly shaped, sometimes confluent, dark brown to black, spindle-shaped, 8-spored asci and allantoid, overlapping, subhyaline ascospores (Trouillas et al. 2011; de Almeida et al. 2016; Dissanayake et al. 2021). In this study, we illustrate *Paraeutypellacitricola* collected from Guizhou Province in China.

#### Paraeutypella citricola

Taxon classificationFungiXylarialesDiatrypaceae

(Speg.). L.S. Dissan., Wijayaw., J.C. Kang & K.D. Hyde, in Dissanayake, Wijayawardene, Dayarathne, Samarakoon & Dai, Biodiversity Data Journal 9: e63864, 14 (2021)

A6C70A8D-3BDC-5C50-A97C-DCEDA081E99D

228646

Fig. 9

≡ Eutypellacitricola Speg., Anal. Mus. nac. Hist. nat. B. Aires 6: 245 (1898)

##### Description.

For description, see Dissanayake et al. (2021)

##### Specimens examined.

China, Guizhou Province, Guiyang City: Aha Lake National Wetland Park (26°20'37.28"N, 108°21'4.34"E), on branches of unidentified plant, 30 August 2020. Altitude: 802 m, S.H. Long, LGS147 (GMB0053, KUN-HKAS 112704, living culture GMBC0053).

##### Additional sequences.

GMB0053 (LSU: 797053, RPB2: MW814898).

##### Notes.

The ITS sequence data were compared by using NCBI and the result showed that it is 100% similar to the ex-type strain (HVVIT07) of *P.citricola*. The morphological features of the new collection are consistent with those described by Dissanayake et al. (2021). This collection is identified as a *P.citricolca*, based on morphological and molecular data.

**Figure 9.**
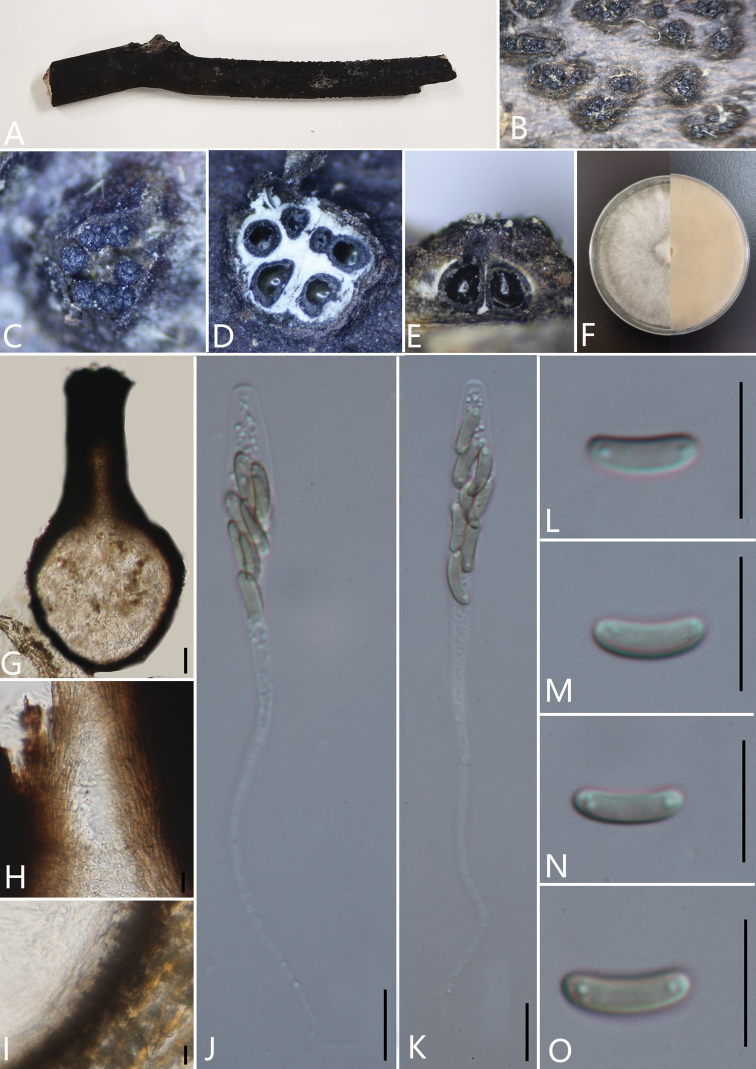
*Paraeutypellacitricola* (GMB0053) **A** stromata on host substrate **B, C** stromata on host **D** transverse section through ascostroma **E** vertical section through ascostroma **F** culture on PDA**G** section through the ascostroma **H** ostiolar canal **I** peridium **J–K** ascospores **L–O** asci. Scale bars: 40 μm (**G**); 10 μm (**H–O**).

## Discussion

In this study, one new genus, three new species, two new records from China, a novel combination and two known species were reported from karst areas of China. We used molecular data to delimit the species of Diatrypaceae. The new genus *Pseudodiatrype* is morphologically similar to *Allodiatrype* and *Diatrype*, but distinct in the size of stromata, number of ascomata and colour of endostroma; it also formed a distinct branch in the phylogenetic analyses (Fig. 1). *Diatrypeoregonensis* was transferred to *Diatrypellaoregonensis* based on the phylogenetic analyses. Based on phylogenetic analyses, *Diatrypellapseudooregonensis* was introduced as an 8-spored species of *Diatrypella*.

Our phylogenetic analyses, based on ITS and β-tubulin, agree with the previous studies (Acero et al. 2004; Trouillas et al. 2011; Mehrabi et al. 2015, 2016; de Almeida et al. 2016; Shang et al. 2017; Dissanayake et al. 2021; Zhu et al. 2021). However, several genera are not monophyletic;for example, *Cryptosphaeria*, *Diatrype*, *Diatrypella*, and *Eutypa*. The identification of species of Diatrypaceae has been a problem due to the polyphyletic generic concepts based on the features of the stromata in early research (Fries 1823). Recently, new approaches have been proposed for classifying Diatrypaceae. Acero et al. (2004) proposed to classify them by ITS sequence-based phylogenetic analyses, while Carmarán et al. (2006) suggested that the identification should be based on the morphology of the asci. However, due to the lack of type specimens, the lack of β-tubulin sequence and polyphyletic origins have resulted in molecular data that correlate poorly with morphological criteria used to delineate genera and species within the Diatrypaceae (Acero et al. 2004). Moreover, Acero et al. (2004) has mentioned that *Diatrypellaquercina* should be placed in the genus *Diatrype* despite its polysporous asci since the molecular data placed *Diatrypellaquercina* in the branch of the genus *Diatrype*.

*Diatrype* and *Diatrypella* have morphologically similar verruculose stromata and allantoid ascospores and the polysporous or 8-spored ascus serve as a basis for distinguishing the two genera. However, in phylogenetic analyses, species of these two genera overlap. In this study, we used the phylogenetic analyses as the main basis for classification following Vasilyeva and Stephenson (2005) and Liu et al. (2015). Clade 1 contains *Diatrypellaverruciformis* which is the type species of *Diatrypella*, of which *Diatrypellapseudooregonensis*, *Diatrypellaoregonensis* have 8-spored, and other species in clade 1 have polyspored ascus. Clade 12 contains the *Diatrype* type species *Diatrypedisciformis*, of which *Diatrypeiranensis* and *Diatrypemacrospora* have polyspored ascus, and other species in clade 12 have 8-spored ascus. Hence, we concluded that the number of ascospores in each ascus cannot be used as a criterion for distinguishing *Diatrypella* from *Diatrype*.

The phylogenetic tree shows that the classification of Diatrypaceae is confusing. Members of *Diatrypella* (*D.favacea*, *D.hubeiensis*, *D.pulvinata* and *D.yunnanensis*) cluster with *Diatrypepalmicola* and *Diatrypelancangensis*.Maybe this clade should be identified as a new genus. We will discuss its classification status after more strains, more gene sequences and new taxonomic features are collected. Some species of *Diatrypella* (*D.iranensis* and *D.macrospora*) which have polysporous ascus are placed between species of *Diatrype*, and they are transferred to *Diatrypeiranensis* and *Diatrypemacrospora* by Zhu et al. (Zhu et al. 2021). *Diatrypeenteroxantha* is often derived from the sister clade of *Allodiatrype* rather than the *Diatrype* clade. Additionally, *Eutypamicroasca* (BAFC51550) clusters with *Peroneutypa* species (Clade 17). The above-mentioned confusion also showed in the original publication and other recent studies (Grassi et al. 2014; Mehrabi et al. 2016; Shang et al. 2018; Hyde et al. 2019; Phookamsak et al. 2019; Konta et al. 2020). Therefore, addressing the taxonomic confusion of this family requires a re-examination of older taxa, based on morphological studies, epitypification and multi-gene phylogenetic analyses (Ariyawansa et al. 2014).

## Supplementary Material

XML Treatment for Diatrype

XML Treatment for Diatrype lancangensis

XML Treatment for Pseudodiatrype

XML Treatment for Pseudodiatrype hainanensis

XML Treatment for Diatrypella

XML Treatment for Diatrypella pseudooregonensis

XML Treatment for Diatrypella vulgaris

XML Treatment for Diatrypella oregonensis

XML Treatment for Allodiatrype

XML Treatment for Allodiatrype thailandica

XML Treatment for Neoeutypella

XML Treatment for Neoeutypella baoshanensis

XML Treatment for Eutypa

XML Treatment for Eutypa cerasi

XML Treatment for Paraeutypella

XML Treatment for Paraeutypella citricola
